# The GAD-7 and the PHQ-8 exhibit the same mathematical pattern of item responses in the general population: analysis of data from the National Health Interview Survey

**DOI:** 10.1186/s40359-021-00657-9

**Published:** 2021-09-23

**Authors:** Shinichiro Tomitaka, Toshiaki A. Furukawa

**Affiliations:** 1 Department of Health Promotion and Human Behavior, Department of Clinical Epidemiology, School of Medicine/School of Public Health, Kyoto University Graduate, Yoshida Konoe-cho, Sakyo-ku, Kyoto, 606-8501 Japan; 2 Department of Mental Health, Panasonic Health Center, Landic Building 3F, Nishishinbashi 3-8-3, Minato-ku, Tokyo, 105-0003 Japan

**Keywords:** Generalized anxiety disorder, GAD-7, Depressive symptoms, PHQ-8, Item response, Mathematical model

## Abstract

**Background:**

Recent studies have shown that, among the general population, responses to depression-rating scales follow a common mathematical pattern. However, the mathematical pattern among responses to the items of the Generalized Anxiety Disorder-7 (GAD-7) is currently unknown. The present study investigated whether item responses to the GAD-7, when administered to the general population, follow the same mathematical distribution as those of depression-rating scales.

**Methods:**

We used data from the 2019 National Health Interview Survey (31,997 individuals), which is a nationwide survey of adults conducted annually in the United States. The patterns of item responses to the GAD-7 and the Patient Health Questionnaire-8 (PHQ-8), respectively, were analyzed inductively.

**Results:**

For all GAD-7 items, the frequency distribution for each response option (“not at all,” “several days,” “more than half the days,” and “nearly every day,” respectively) was positively skewed. Line charts representing the responses to each GAD-7 item all crossed at a single point between “not at all” and “several days” and, on a logarithmic scale, showed a parallel pattern from “several days” to “nearly every day.” This mathematical pattern among the item responses was identical to that of the PHQ-8. This characteristic pattern of the item responses developed because the values for the “more than half the days” to “several days” ratio were similar across all items, as were the values for the “nearly every day” to “more than half the days” ratio.

**Conclusions:**

Our results suggest that the symptom criteria of generalized anxiety disorder and major depression have a common distribution pattern in the general population.

**Supplementary Information:**

The online version contains supplementary material available at 10.1186/s40359-021-00657-9.

## Background

Generalized anxiety disorder (GAD) is one of the most common mental disorders [1]. Among the general population, its 12-month prevalence and lifetime morbid risk are estimated to be 2.0% and 9.0%, respectively [2, 3]. Several screening instruments have been developed for effectively identifying probable cases of GAD [4, 5]. In particular, the Generalized Anxiety Disorder-7 (GAD-7) is one of the most commonly used tools for GAD screening worldwide [6, 7]. The GAD-7 measures GAD based on the associated symptom criteria listed in the Diagnostic and Statistical Manual of Mental Disorders, Fourth Edition [4]. It is valid and reliable, with a sensitivity of 89%, a specificity of 82%, and a high Cronbach Alpha value (0.9) [8, 9]. The distribution pattern, among the general population, of responses to the GAD-7 symptom criteria, is of interest because, although the diagnosis of GAD is based on responses to these symptom criteria, the mathematical pattern of responses to the GAD-7 items in the context of the general population is currently unknown.

**Fig. 1:**
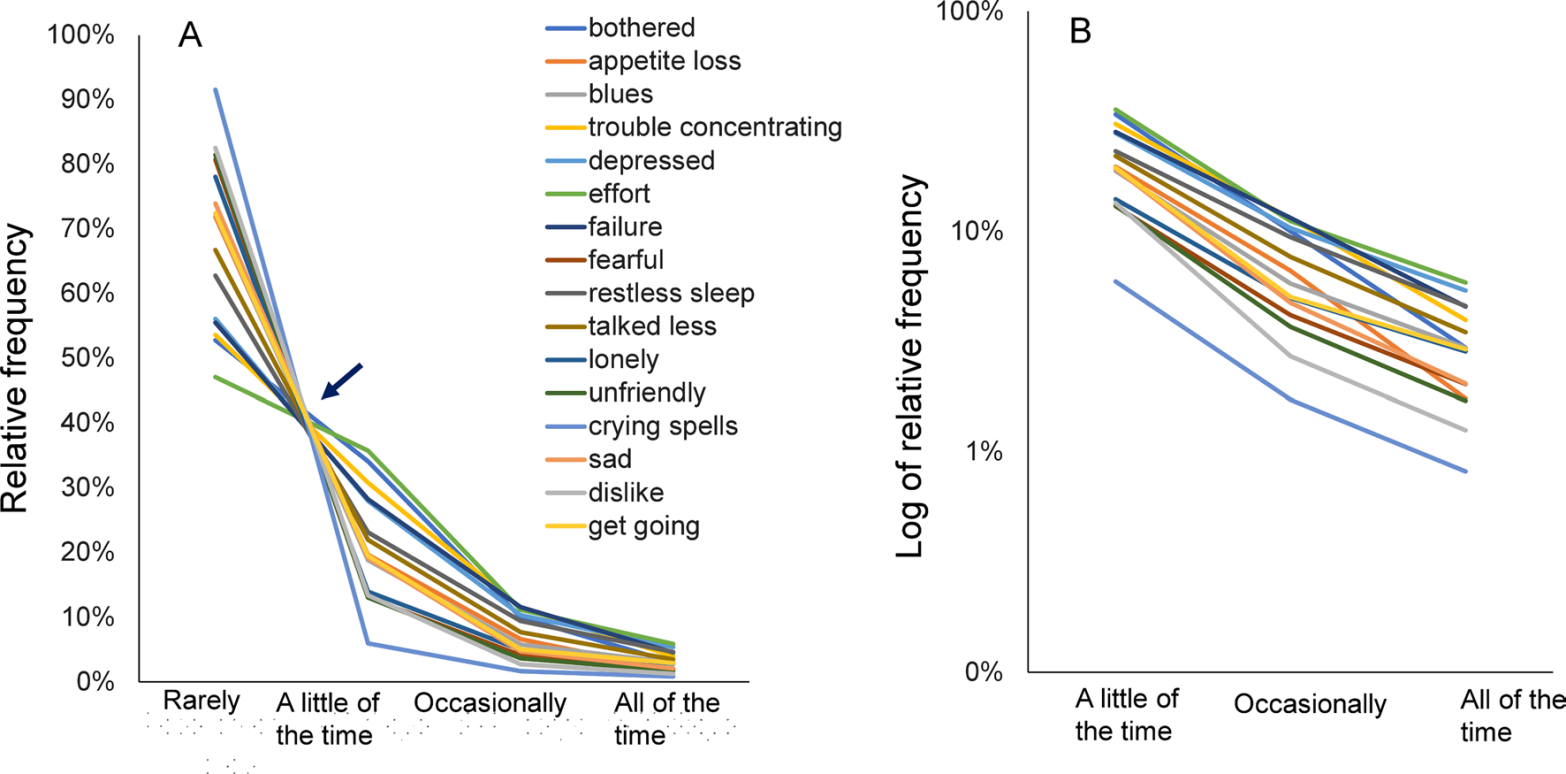
Responses to the 16 depressive-symptom items of the Center for Epidemiologic Studies Depression Scale. Responses to the 16 items are presented using a normal scale (**A**), and a logarithmic scale (**B**). **A** The lines for the item responses appear to cross at a single point between “rarely” and “a little of the time,” whereas they display a converging pattern between “a little of the time” and “all of the time.” **B** On a logarithmic scale, the lines for the 16 items exhibit a parallel linear pattern from “a little of the time” to “all of the time.” Image used under CC-BY license: PLoS ONE, 10.1371/journal.pone.0165928.g001

Major depression is another common mental disorder; among the general population, its 12-month prevalence and lifetime morbid risk are estimated to be 8.6% and 29.9%, respectively [2]. Recent analyses of large-scale national survey data have shown that responses to scale items concerning depressive symptoms exhibit a common mathematical pattern among the general population. In an analysis of data from a Japanese national survey in which the Center for Epidemiologic Studies Depression Scale (CES-D) was administered to members of the general population, the present authors found that responses to 16 depressive-symptom items exhibit a common mathematical pattern among the general population [10, 11]. Figure 1A shows the relative frequency of each response for each of the 16 items. In this figure, it can be seen that the lines cross between “rarely” and “a little of the time,” with most of them meeting at a single point on the graph; then, they begin to converge between “a little of the time” and “all of the time.” Meanwhile, on a logarithmic scale, the converging lines show a parallel pattern from “a little of the time” to “all of the time” (Fig. 1B) [11]. The existence of a common mathematical pattern in responses to depression-rating scale items has been confirmed for a considerable number of nationally representative survey datasets worldwide; for example, CES-D data from the Irish Longitudinal Study on Ageing [12], 12-item General Health Questionnaire data from the Eurobarometer surveys [13], nine-item Patient Health Questionnaire (PHQ-9) data from the National Health and Nutrition Examination Survey in the United States [14], and six-item Kessler Psychological Distress Scale data from the national survey of Midlife Development [15] and the National Health Interview Survey in the United States [16]. This strongly suggests that the reproducibility of the item-response pattern is high. Moreover, it is noteworthy that rating scales other than depression-rating scales do not show such a mathematical pattern among the general population [10].

There are two primary reasons why identifying the mathematical pattern of responses to the GAD-7 is important. First, if the item responses to the GAD-7 and depression-rating scales show the same mathematical pattern, this will contribute to improving understanding of the relationship between GAD-7 and depression-rating scales. In general, the mathematical pattern of a sampling distribution reflects the mechanism by which the variables are generated [17]. Thus, if the GAD-7 and depression-rating scales share a common mathematical pattern in terms of item responses, this will suggest that the symptom criteria of GAD and major depression have a common distribution mechanism. Such a relationship is somewhat likely, as GAD-7 and depression-rating scales are known to be strongly linked; studies have reported moderate to strong correlations between the GAD-7 and depression-rating scales [4, 18, 19]. Moreover, numerous studies have reported high comorbidity of GAD and depression [1, 20–22]. Second, if a mathematical pattern is identified among the item responses to the GAD-7, this would help conduct statistical procedures. Parametric statistics that assume normality are widely used to analyze rating-scale data [23]; however, there is currently no evidence that the item responses to the GAD-7 follow a normally distributed latent variable. Thus, to conduct statistical procedures regarding the GAD-7, it is essential to determine whether the variables feature a mathematical pattern [24].

Generally, larger datasets enable researchers to better analyze sampling distribution patterns. The National Health Interview Survey (NHIS) is conducted annually in the United States and is designed to provide nationally representative estimates for a range of health status variables [25]. In 2019, the NHIS employed the GAD-7 and the eight-item Patient Health Questionnaire (PHQ-8) for the first time in its long history [26]. The PHQ-8 has good validity and reliability for detecting major depression, with a sensitivity of 88%, a specificity of 88%, and a high Cronbach’s Alpha value (0.89) [27]. As a result of the large sample size and limited selection bias involved, the GAD-7 and PHQ-8 data collected through the NHIS are suitable for clarifying the aforementioned issue regarding determining the mathematical pattern of the GAD-7.

Using data from the 2019 edition of the NHIS, the present study investigated the respective mathematical patterns of the item responses to the GAD-7 and PHQ-8 when administered to members of the general population. In particular, we sought to determine whether responses to anxiety-rating scales exhibit the same mathematical pattern as those to depression-rating scales. Furthermore, we built a distribution model for the item responses for the GAD-7.

## Methods

### Dataset

We used data from the 2019 edition of the NHIS. The NHIS, which is conducted annually by the National Center for Health Statistics, is designed to survey the health of the general population of the United States and obtain nationally representative estimates of certain health variables [28]. The NHIS targets the civilian noninstitutionalized population of the United States. A sample of households is created, and from each participating family, one “sample adult” aged 18 years or older is randomly selected and invited to participate in the survey. In 2019, the final response rate was 59.1% [28]. The datasets analyzed during the present study are available from the NHIS repository [25]; NHIS data are available to researchers worldwide.

The sample comprised 31,997 respondents (ages 18–19 years: n = 530 [male: n = 263]; ages 20–29 years: n = 3944 [male: n = 1,881]; ages 30–39 years: n = 5178 [male: n = 2393]; ages 40–49 years: n = 4656 [male: n = 2238]; ages 50–59 years: n = 5,282 [male: n = 2573]; ages 60–69 years: n = 5921 [male: n = 2707]; ages 70–79 years: n = 4072 [male: n = 1766]; ages 80 years and older: n = 2414 [male: n = 912]). The sociodemographic characteristics of the 2019 NHIS sample are reported in detail elsewhere [26].

### Ethics statement

The present study used de-identified data that are available to the public. The ethics committees of Kyoto University and Panasonic Health Center do not consider the analysis of de-identified public data to represent research of human subjects. These committees ruled that institutional review board approval was unnecessary for the present research.

### Measures

The 2019 NHIS questionnaires included the GAD-7 and the PHQ-8. The GAD-7 comprises seven items that are based on the symptom criteria of GAD listed in the DSM-5 [4]. In the 2019 NHIS, respondents were asked how often they had experienced each symptom during the past two weeks. Each item was self-rated using four-point response options: “not at all,” “several days,” “more than half the days,” and “nearly every day,” which were scored as 0, 1, 2, and 3, respectively. Meanwhile, the PHQ-8 comprises eight items and is designed to assess major depressive disorder, also based on the DSM-5’s associated symptom criteria [29]. The PHQ-8 is very similar to the PHQ-9 but omits item 9 of the PHQ-9 (“How often have you been bothered by thoughts that you would be better off dead or hurting yourself in some way?”). PHQ-8 items are answered using the same response scale as that used for the GAD-7.

### Analysis

First, we analyzed the pattern of item responses to the PHQ-8 to identify whether any common characteristics were present. As the 2019 NHIS was a survey with a complex design, analyses of item response frequencies were weighted by survey-specific weights which made the sample representative of the target population [8]. Previous studies have reported that, when a self-report scale for measuring depression is administered among the general population, the ratios between consecutive response options remain similar across all items in the scale, except for the option at the lower end of the score range [30]. Thus, the respective ratios of “more than half the days” to “several days” and “nearly every day” to “more than half the days” were calculated for all eight items of the PHQ-8. Thereafter, we graphically analyzed the mathematical pattern of item responses to the PHQ-8. Similarly, we analyzed the respective ratios of “more than half the days” to “several days,” and “nearly every day” to “more than half the days” for the GAD-7. Thereafter, we graphically investigated whether the item responses to the GAD-7 followed the same pattern as those of the PHQ-8. These analyses were performed by sex. Correlations were examined between the GAD-7 and the PHQ-8.

Based on the consequent finding that the values for the “more than half the days” to “several days” ratio and the values for the “nearly every day” to “more than half the days” ratio were similar across all items of the GAD-7, we built an inductive model of the item responses to the GAD-7. Analyses were conducted using IBM SPSS complex samples for Windows, version 27 (IBM Corp., Armonk, N.Y., USA).

## Results

### Demographic characteristics of the participants

Of the 31,997 respondents, those who did not respond to all items of the PHQ-8 and the GAD-7 (3.3%, n = 1043) were excluded from this analysis. The final sample consequently comprised 30,954 respondents (14,262 males; ages 18–19: n = 515 [male, n = 254]; ages 20–29: n = 3840 [male, n = 1841]; ages 30–39: n = 5036 [male, n = 2327]; ages 40–49: n = 4512 [male, n = 2175]; ages 50–59: n = 5107 [male; n = 2479]; ages 60–69: n = 5741 [male, n = 2628]; ages 70–79: n = 3933 [male, n = 1693]; and age 80 or older: n = 2270 [male, n = 865]).

### Correlation among the 15-item scores of the GAD-7 and the PHQ-8

Although to varying degrees, all items of the PHQ-8 and the GAD-7 had moderate-to-strong positive Spearman’s correlations (ρ = 0.30–0.71) (Additional file 1: Table S1). The Pearson’s correlation between the total GAD-7 scores and the PHQ-8 scores was 0.79.

### PHQ-8 item responses

Table 1 displays the response rates for the PHQ-8 items. A common tendency was observed for all seven items, with the frequency being highest for “not at all,” decreasing from “not at all” to “more than half the days,” and increasing from “more than half the days” to “nearly every day.” There were no exceptions to this tendency. The rates of “more than half the days” to “several days” and “nearly every day” to “more than half the days” were 0.23 ± 0.04, and 1.52 ± 0.17, respectively. The standard deviations of the two rates were rather small when compared with the averages, which suggests that the two rates were similar, to some extent, across the eight items.

**Table 1:** Item responses to the PHQ-8

Item	Item responses				Rate of more than half the days to several days	Rate of nearly every day to more than half the days
	Not at all (%)	Several days (%)	More than half the days (%)	Nearly every day (%)		
1. Loss of interest or pleasure	83.2	11.1	2.4	3.3	0.21	1.38
2. Depressed mood	83.4	11.9	2.1	2.6	0.18	1.27
3. Sleep disturbances	68.5	18.4	4.7	8.4	0.26	1.79
4. Anergia or fatigability	60.0	25.7	5.4	8.8	0.21	1.61
5. Appetite disturbances	82.7	10.3	2.8	4.2	0.27	1.52
6. Low self-esteem	86.8	8.9	1.8	2.5	0.20	1.42
7. Concentration difficulties	88.1	7.4	1.7	2.8	0.23	1.69
8. Psychomotor symptoms	93.7	3.7	1.0	1.5	0.28	1.47
Average	80.8	12.2	2.7	4.3	0.23 ± 0.04	1.52 ± 0.17

Each of the eight items was scored using a four-point scale: 0 (indicating “not at all”), 1 (“several days”), 2 (“more than half the days”), and 3 (“nearly every day”). Average rate data are presented as mean ± standard deviation.

**Fig. 2:**
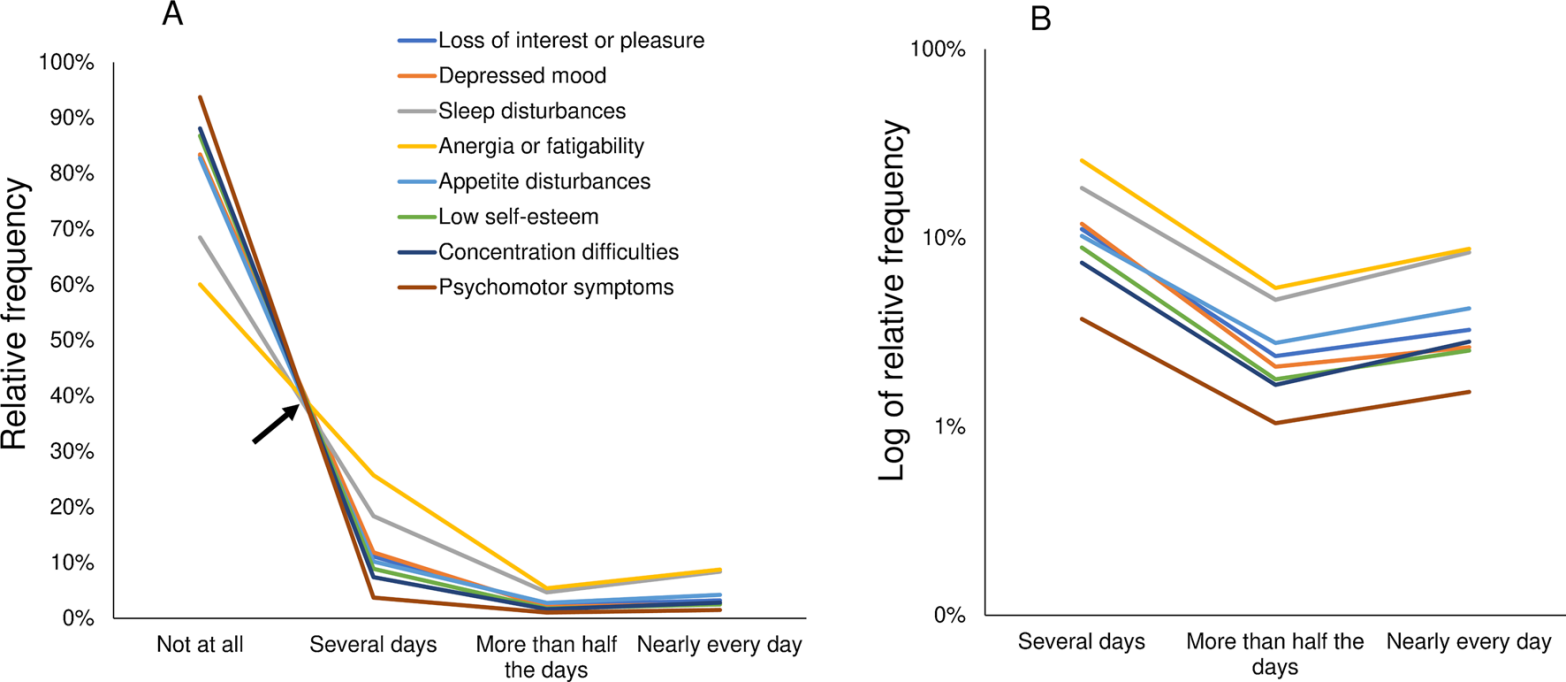
Responses to the eight depressive-symptom items of the Patient Health Questionnaire-8. The responses to the eight items are presented using a normal scale (**A**), and a logarithmic scale (**B**). **A** As indicated by the arrow, the line graphs of the eight items appear to cross at a single point between “not at all” and “several days.” The lines decrease in synchrony from “several days” to “more than half the days,” before increasing in synchrony from “more than half the days” to “nearly every day.” **B** The lines show parallel fluctuation from “several days” to “nearly every day.”

To assess the pattern among the item responses for the PHQ-8, line graphs representing the response frequencies for each item were plotted onto a single graph (Fig. 2). As indicated by the arrow shown in Fig. 2A, the lines for all eight items appeared to cross at a single point between “not at all” and “several days.” Conversely, from “several days” to “more than half the days” the lines decreased in synchrony, before increasing in synchrony from “more than half the days” to “nearly every day.”

When presented on a logarithmic scale (Fig. 2B), the lines showed a generally parallel pattern from “several days” to “nearly every day.” Mathematically, this parallelism of the eight lines on the logarithmic scale reflects the similarity of the values for the “more than half the days” to “several days” ratio across the eight items, and of the values for the “nearly every day” to “more than half the days” ratio across the items, respectively [30]. This supports the abovementioned observation that these ratios were similar, to some extent, among all items (Table 1). Taken together, the graphical analysis confirmed that the item responses exhibited a common pattern across the eight items.

### Item responses to the GAD-7

Table 2 shows the item response rates for the GAD-7. The responses for all seven items showed a similar pattern, with the frequency being highest for “not at all,” decreasing from “not at all” to “more than half the days,” and increasing from “more than half the days” to “nearly every day”; this was consistent with the rate distribution among the PHQ-8 items. The respective rates of “more than half the days” to “several days” and “nearly every day” to “more than half the days” were 0.19 ± 0.02, and 1.55 ± 0.25, respectively, also similar to those of the PHQ-8 (0.23 ± 0.04, and 1.52 ± 0.17, respectively). Moreover, the standard deviations of the two rates were rather small in comparison to the averages, again consistent with the results of the PHQ-8.

**Table 2:** Item responses to the GAD-7

Item	Item responses (%)				Rate of more than half the days to several days	Rate of nearly every day to more than half the days
	Not at all (%)	Several days (%)	More than half the days (%)	Nearly every day (%)		
1. Feeling nervous, anxious, or on edge	76.5	16.8	2.9	3.8	0.17	1.30
2. Not being able to stop or control worrying	83.3	10.5	2.3	3.9	0.21	1.74
3. Worrying too much about different things	76.3	16.3	2.8	4.6	0.17	1.64
4. Trouble relaxing	80.7	12.6	2.5	4.2	0.20	1.64
5. Being so restless that it is hard to sit still	88.6	7.2	1.5	2.7	0.20	1.87
6. Becoming easily annoyed or irritable	74.9	18.1	3.3	3.8	0.18	1.15
7. Feeling afraid as if something awful might happen	87.8	8.2	1.6	2.4	0.20	1.49
Average	81.1	12.8	2.4	3.6	0.19 ± 0.02	1.55 ± 0.25

Each of the seven items is scored on a four-point scale: 0 (indicating “not at all”), 1 (“several days”), 2 (“more than half the days”), and 3 (“nearly every day”). Average rate data are presented as mean ± standard deviation.

**Fig. 3:**
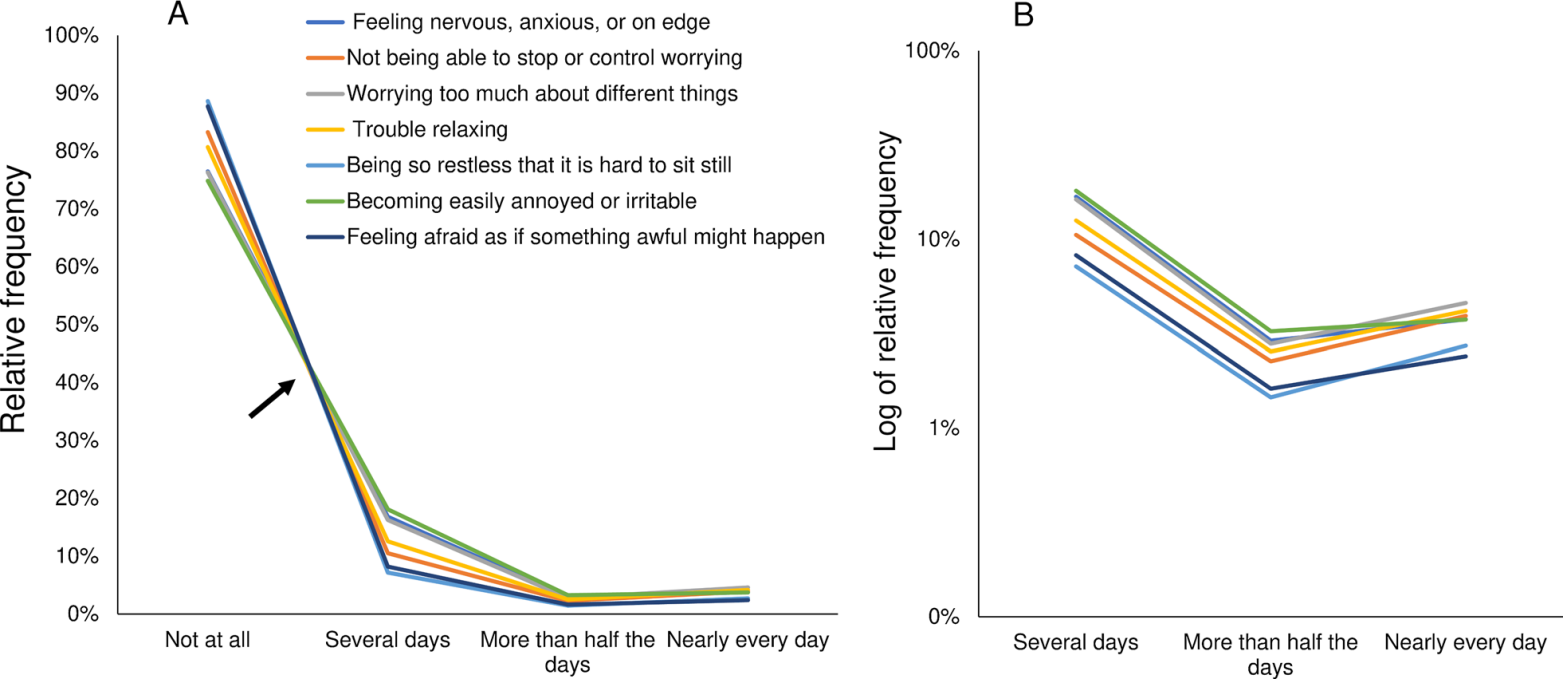
Responses to the seven items of the Generalized Anxiety Disorder-7. Responses to the seven items are presented using a normal scale (**A**), and a logarithmic scale (**B**). **A** As indicated by the arrow, the line graphs of the seven items appear to cross at a single point between “not at all” and “several days.” The lines decrease in synchrony from “several days” to “more than half the days,” before increasing in synchrony from “more than half the days” to “nearly every day.” **B** The lines show a parallel pattern from “several days” to “nearly every day.”

To identify the patterns of the item responses, line graphs representing the response frequencies for each item were plotted on the same scale (Fig. 3). Consistent with the results of the PHQ-8, the responses exhibited a common pattern across the seven items of the GAD-7. As indicated by the arrow shown in Fig. 3A, the lines for the eight items appeared to cross at a single point between “not at all” and “several days.” The lines for the seven items then decreased in synchrony from “several days” to “more than half the days,” before increasing in synchrony from “more than half the days” to “nearly every day.” When compared to the line graphs for the responses to the PHQ-8 (Fig. 2A), the seven lines representing the responses to the GAD-7 seemed to overlap to a greater extent (Fig. 3A).

When presented on a logarithmic scale, the lines for each item response showed a generally parallel pattern from “several days” to “nearly every day” (Fig. 3B). The gradient of the linear patterns of item responses decreases from “several days” to “more than half the days,” and then increases from “more than half the days” to “nearly every day.” While, between “several days” and “more than half the days,” the lines appear to follow an almost perfect parallel pattern, between “more than half the days” and “nearly every day” the pattern is less parallel. These observations accord with the finding that the standard deviation of the ratio of “more than half the days” to “several days” (0.02) was smaller when compared to that of “nearly every day” to “more than half the days” (0.25).

### Common pattern in all responses to the PHQ-8 and GAD-7

**Fig. 4:**
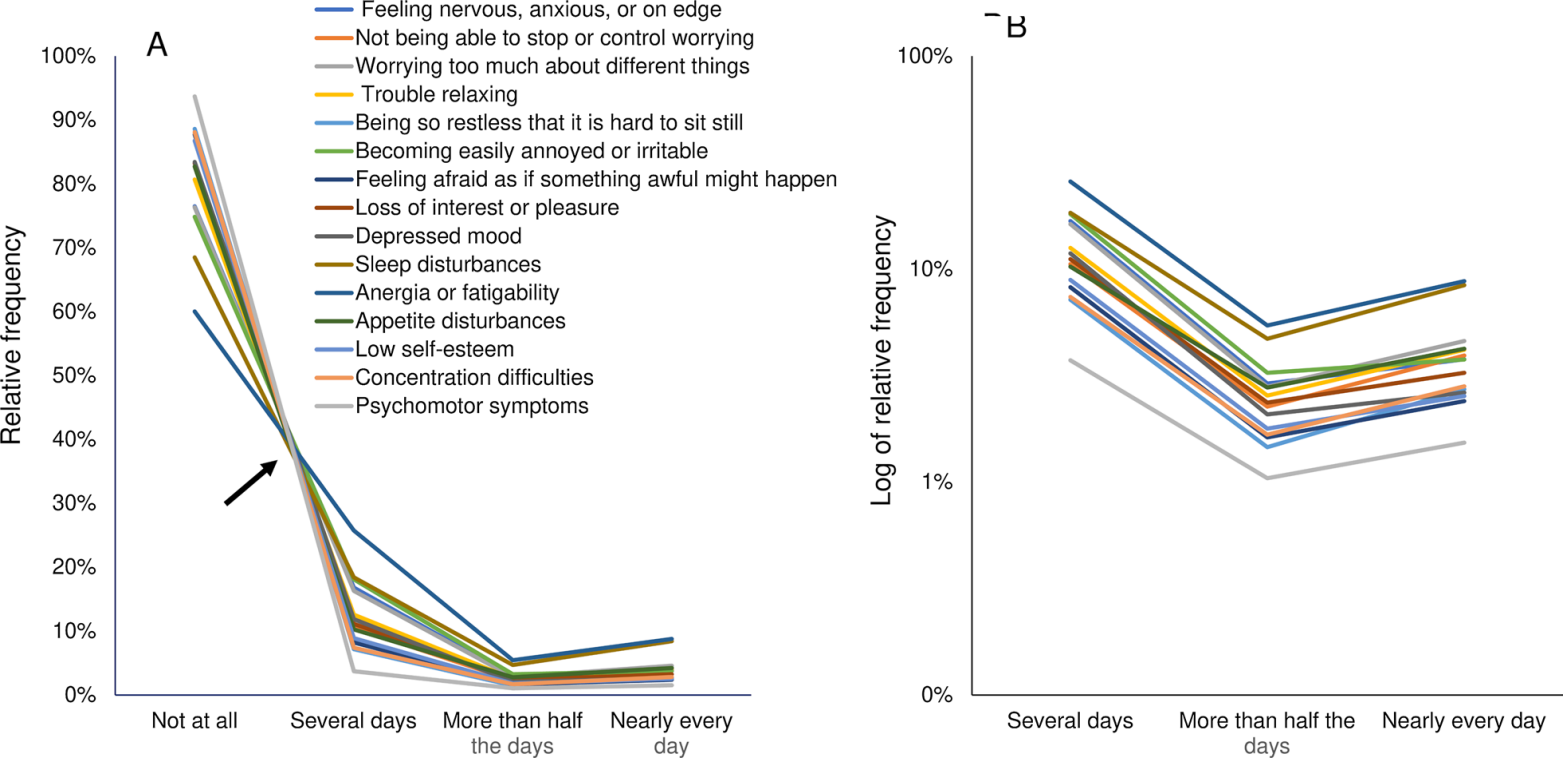
Responses to the 15 items of the Patient Health Questionnaire-8 and the Generalized Anxiety Disorder-7. Responses to the 15 items are presented using a normal scale (**A**), and a logarithmic scale (**B**). **A** As indicated by the arrow, the line graphs of the seven items appear to cross at a single point between “not at all” and “several days.” The lines decrease in synchrony from “several days” to “more than half the days,” before increasing in synchrony from “more than half the days” to “nearly every day.” **B** The lines show parallel fluctuation from “several days” to “nearly every day.”

To confirm whether all item responses to the PHQ-8 and GAD-7 show the same mathematical pattern, all of these item responses were plotted on the same scale (Fig. 4). As indicated by the arrow shown in Fig. 4A, the lines for all 15 items appeared to cross at a single point between “not at all” and “several days.” Conversely, the lines decreased in synchrony from “several days” to “more than half the days,” before increasing in synchrony from “more than half the days” to “nearly every day.”

When presented on a logarithmic scale, the lines for the 15 item responses showed a generally parallel pattern from “several days” to “nearly every day” (Fig. 4B). Taken together, these graphs indicated that all responses to the PHQ-8 and GAD-7 show a common mathematical pattern.

To confirm whether all item responses to the PHQ-8 and GAD-7 showed the same mathematical pattern regardless of sex, we performed graphical analyses. For both males (Additional file 2: Fig. S1) and females (Additional file 3: Fig. S2), the item responses of the GAD-7 and the PHQ-8 followed the same mathematical distribution.

### Mathematical model of the item responses for the GAD-7 and PHQ-8

Based on the finding that the values for the “more than half the days” to “several days” ratio were similar across all items of the PHQ-8 and GAD-7, and that the values for the “nearly every day” to “more than half the days” ratio were also similar across these items, we built a mathematical model for the item responses for these scales.

**Fig. 5:**
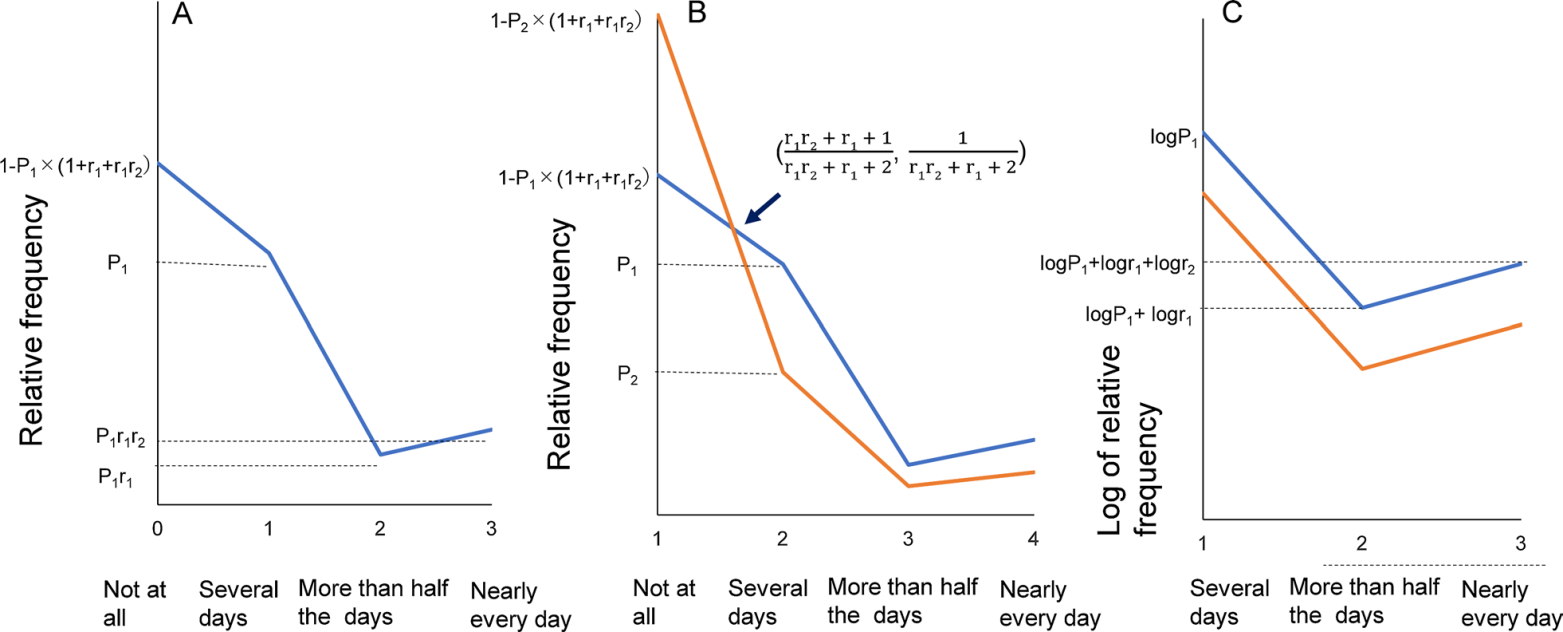
Mathematical model for the item responses for Generalized Anxiety Disorder-7. **A** The blue line illustrates the pattern of the model of the item responses for such scales. When the relative frequency of “several days,” the ratio of “more than half the days” to “several days,” and the ratio of “nearly every day” to “more than half the days” are presented as P_1_, r_1,_ and r_2_, respectively, the relative frequencies of “not at all,” “several days,” “more than half the days,” and “nearly every day” are expressed as 1 − P_1_ × (1 + r_1_ + r_1_r_2_), P_1_, P_1_r_1_, and P_1_r_1_r_2_, respectively. **B** The relative frequencies of “not at all,” “several days,” “more than half the days,” and “nearly every day” for another item (the red line) are expressed as 1 – P_2_ × (1 + r_1_ + r_1_r_2_), P_2_, P_2_r_1_, and P_2_r_1_r_2_, respectively. According to the calculation, the intersection between “not at all” and “several days,” is expressed as follows: (x, y) = ($\frac{\text{r}1\text{r}2 + \text{r}1 + 1}{\text{r}1\text{r}2 + \text{r}1 + 2}$, $\frac{1}{\text{r}1\text{r}2 + \text{r}1 + 2}$). **C** A line chart model of the item responses between “several days” and “nearly every day” on a logarithmic scale. On the logarithmic scale, the relative frequencies of “several days,” “more than half the days,” and “nearly every day” for the blue line are expressed as logP_1_, logP_1_ + logr_1_, and logP_1_ + logr_1_ + logr_2,_ respectively. Consequently, the slopes from “several days” to “more than half the days,” and from “more than half the days” to “nearly every day” are expressed as logr_1_ and logr_2_, respectively

The blue line shown in Fig. 5A illustrates the pattern of the model of the item responses for such scales. For a four-point scale such as the GAD-7, when the relative frequency of “several days,” the ratio of “more than half the days” to “several days,” and the ratio of “nearly every day” to “more than half the days” are presented as P_1_, r_1,_ and r_2_, respectively, the relative frequencies of “not at all,” “several days,” “more than half the days,” and “nearly every day” are expressed as 1 − P_1_ × (1 + r_1_ + r_1_r_2_), P_1_, P_1_r_1_, and P_1_r_1_r_2_, respectively (Fig. 5A).

As shown in Fig. 5B, the relative frequencies of “not at all,” “several days,” “more than half the days,” and “nearly every day” for another item (the red line) are expressed as 1 – P_2_ × (1 + r_1_ + r_1_r_2_), P_2_, P_2_r_1_, and P_2_r_1_r_2_, respectively. According to the calculation, the intersection between “not at all” and “several days,” is expressed as follows: (x, y) = ($\frac{\text{r}1\text{r}2 + \text{r}1 + 1}{\text{r}1\text{r}2 + \text{r}1 + 2}$, $\frac{1}{\text{r}1\text{r}2 + \text{r}1 + 2}$). The intersection point is expressed by r_1_ and r_2_ only. Consequently, regardless of the value of P_1_ or P_2_, all of the lines cross at a single point between “not at all” and “several days.” According to the model of the item responses, it is natural that the lines of all 15 items appear to cross at a single point between “not at all” and “several days” (Fig. 5A), because the respective rates of “more than half the days” to “several days” (r_1_) and “nearly every day” to “more than half the days” (r_2_) were similar across the PHQ-8 and the GAD-7 (Tables 1, 2).

Figure  5C shows a line chart model of the item responses between “several days” and “nearly every day” on a logarithmic scale. On the logarithmic scale, the relative frequencies of “several days,” “more than half the days,” and “nearly every day” for the blue line are expressed as logP_1_, logP_1_ + logr_1_, and logP_1_ + logr_1_ + logr_2,_ respectively. Consequently, the slopes from “several days” to “more than half the days,” and from “more than half the days” to “nearly every day” are expressed as logr_1_ and logr_2_, respectively. It is noteworthy that the slopes on the logarithmic scale are expressed by r_1_ and r_2_ only. Therefore, regardless of the value of P_1_ or P_2_, all the lines show a parallel pattern between “several days” and “nearly every day” on a logarithmic scale.

## Discussion

In this study, we found a common mathematical pattern among responses to the seven GAD-7 items when this scale is administered to the general population. The pattern was characterized by the lines crossing at a single point between the responses “not at all” and “several days,” and, on a logarithmic scale, showing a parallel pattern from “several days” to “nearly every day.” This pattern was consistent with the corresponding trend for the PHQ-8. A mathematical model of the item responses showed that the characteristic pattern of the item responses developed because the values for the “more than half the days” to “several days” ratio were similar across all items, as were the values for the “nearly every day” to “more than half the days” ratio.

For both the GAD-7 and PHQ-8, differing patterns were observed between the trends for the lower end of the response options and those for the remaining options; this is consistent with findings for the CES-D (Figs. 1, 2, 3). A possible reason for this finding is the psychological process underlying how each symptom item is rated [10]. In general, assessment of each symptom criterion is conducted in two stages. In the first stage, each respondent assesses whether the given symptom criterion is present. If the degree of each symptom criterion does not reach the threshold at which the respondent notices the symptom, it is categorized as “not at all.” However, if the degree of the symptom criterion reaches or exceeds this threshold, it is categorized using the remaining response options, such as “several days,” “more than half the days,” and “nearly every day.” This two-stage process implies that “not at all” corresponds to the under-threshold range, while the remaining degree-adverb options correspond to the over-threshold range. If each of the remaining response options corresponds to a specific proportion of the over-threshold range, the item responses should show different patterns between the response option at the lower end and the remaining options [30]. Further research should focus on how each of the remaining response options can correspond to a specific proportion of the over-threshold range.

The item responses for the GAD-7 and PHQ-8 exhibit the same mathematical pattern. This suggests that symptoms of GAD and major depression share a common distribution mechanism. Supporting this hypothesis, the 15 items of the PHQ-9 and the GAD-7 had moderate-to-strong positive correlations in this study. Moreover, numerous studies have reported that GAD and depression have high comorbidity [1, 20–22]. The present authors have previously shown that a characteristic pattern of item responses occurs only when a general trait of the variables in question follows an exponential distribution [30].

When compared to the graph for the PHQ-8 (Fig. 2A), the seven lines in the graph representing the responses to the GAD-7 overlapped to a greater extent (Fig. 3A). In fact, for the GAD-7 the frequency of “not at all” ranged from 74.9 to 88.6% (Table 2), whereas for the PHQ-8 the frequency of “not at all” ranged from 60.0 to 93.7% (Table 1). This difference between the GAD-7 and the PHQ-8 regarding the range of “not at all” may reflect a difference in how the items of each scale were selected by the scale developers. The symptom criteria measured in the GAD-7 were selected by rank ordering items based on their respective correlations with the total score for a larger scale that reflected all of the symptom criteria for GAD contained in the Diagnostic and Statistical Manual of Mental Disorders, Fourth Edition [4]. In contrast, the PHQ-8 includes a wide range of symptoms, such as psychological, somatic, and social symptoms. The criterion symptoms of the PHQ-8 derive from the Feighner criteria for depression, which emphasize consideration of the multifaceted nature of symptoms rather than rank ordering of the correlations of each symptom [31].

On a logarithmic scale, the lines for the PHQ-8 and the GAD-7 items showed a stronger parallel pattern between “several days” and “more than half the days” when compared to the pattern between “more than half the days” and “nearly every day” (Figs. 2, 3). This finding accords with those of previous investigations involving the PHQ-9 and the CES-D [10, 14]. A possible explanation for this difference is the sample sizes in question; supporting this possible explanation is the fact that the relative frequencies of “more than half the days” and “nearly every day” were much smaller than that of “several days.”

For both sexes, the item responses for the GAD-7 and PHQ-8 exhibited the same mathematical pattern. Previous analyses have reported that the item responses for depression rating scales show the same mathematical pattern regardless of age and nationality [13, 32]. Taken together, item responses on the GAD-7 and depression rating scales may follow the same characteristic pattern regardless of demographic factors, such as sex, age, and nationality. However, it is unknown whether the GAD-7 and depressive symptom scales follow the same mathematical distribution in a clinical population. A future study with more focus on a clinical population is therefore suggested.

This study has several limitations. First, we did not investigate whether the findings were generalizable to symptom criteria associated with other anxiety disorders. Extensive additional research is necessary to generalize the findings to such symptoms. Second, this study lacks quantification of the goodness of fit of the model presented. When determining the fit of established unitary models (i.e., normal, linear, and quadratic models), established methods can be used. However, the present model is unique and complicated. Moreover, a unified descriptor for interpreting the goodness of fit does not exist yet. Therefore, we were unable to describe the degree of the present model’s fit using unified descriptors, such as “slightly,” “moderately,” and “strongly.” Further research is necessary to quantify the fit of the present model. Third, because of the cross-sectional nature of the data, we could not examine the temporal sequencing of the distribution pattern of depression and anxiety symptoms. Several studies suggest that anxiety disorders tend to temporally precede depression [33–35]. Further longitudinal studies are necessary to clarify the temporal sequencing of the distribution pattern of depression and anxiety symptoms. Finally, an important limitation of this research is the representativeness of the study sample; we performed a complete-case analysis which could have induced response bias. Another strategy for handling missing data is multiple imputation, which simulates the missing data based on theory. However, most multiple imputation softwares assume that data are normally distributed. Since the variables of our data are not normally distributed, there is the statistical concern that multiple imputation can induce bias [36]. In addition, the final response rate of the NHIS survey was 59.1%. This could also have induced bias because the characteristics of non-responders may differ from responders [37].

Despite the above limitations, this study also has several strengths. First, the use of data from the NHIS meant that a large sample size with limited selection bias was analyzed. Second, although the present study employed a simple analysis approach (visualization using line charts), it enabled us to identify a complex pattern of item responses; graphical analysis is useful for exploratory data analysis of complex models [38]. Third, our observation of a mathematical pattern of item responses in data representing a large sample size is noteworthy because distributional models are needed to conduct statistical procedures. Finally, this is the first report on the mathematical pattern among item responses to the GAD-7 when this scale is administered to the general population. The fact that the item responses to the GAD-7 and the PHQ-8 exhibit the same mathematical distribution provides additional insight into the mechanism of these scales. From the viewpoint of public health, the distribution pattern of item responses on the GAD-7 is necessary to analyze the distribution of anxiety conditions in defined populations. The mathematical model for item responses on the GAD-7 enables us to easily describe the distribution of anxiety symptoms with parameters. Moreover, as noted previously, the observed distribution patterns provide evidence that item responses on the GAD-7 follow a non-normal distribution in the general population, suggesting that statistical methods assuming normality require careful consideration when analyzing such data. More research should be undertaken to further explore how item responses to such scales show a common mathematical pattern among the general population.

## Conclusions

The findings of this study provide evidence that there is a common mathematical pattern among the item responses to the GAD-7. Given that the item responses to the GAD-7 exhibit the same mathematical distribution as those of the PHQ-8, we conjecture that symptoms of GAD and major depression share a common distribution mechanism. This finding that the item responses to the GAD-7 and the PHQ-8 exhibit the same mathematical distribution provides further insight into the relationship between anxiety and depression.

## Supplementary Information

**Additional file 1.** Correlation among the 15-item scores of the GAD-7 and the PHQ-8.

**Additional file 2: Fig. S1.** Responses to the 15 items of the Patient Health Questionnaire-8 and the Generalized Anxiety Disorder-7 in males. Responses by males to the 15 items are presented using a normal scale (**A**) and a logarithmic scale (**B**). **A** As indicated by the arrow, the line graphs of the 15 items appear to cross at a single point between “not at all” and “several days.” The lines decrease in synchrony from “several days” to “more than half the days,” before increasing in synchrony from “more than half the days” to “nearly every day.”

**Additional file 3: Fig. S2.** Responses to the 15 items of the Patient Health Questionnaire-8 and the Generalized Anxiety Disorder-7 in females. Responses by females to the 15 items are presented using a normal scale (**A**) and a logarithmic scale (**B**). **A** As indicated by the arrow, the line graphs of the 15 items appear to cross at a single point between “not at all” and “several days.” The lines decrease in synchrony from “several days” to “more than half the days,” before increasing in synchrony from “more than half the days” to “nearly every day.”

## Data Availability

The datasets analyzed in the present study are available from the National Health Interview Survey’s official repository: https://www.cdc.gov/nchs/nhis/nhis_2019_data_release.htm.

## Abbreviations

CES-D: Center for Epidemiologic Studies Depression Scale
DSM-5: Diagnostic and Statistical Manual of Mental Disorders, Fifth Edition
GAD: generalized anxiety disorder
GAD-7: Generalized Anxiety Disorder Scale
NHIS: National Health Interview Survey
PHQ: patient health questionnaire
