# Supplementary figures and images for: The GAD-7 and the PHQ-8 exhibit the same mathematical pattern of item responses in the general population: analysis of data from the National Health Interview Survey
Source: BMC Psychol. 2021 Sep 23;9:149. doi: 10.1186/s40359-021-00657-9 (PMC8461873; doi:10.1186/s40359-021-00657-9)

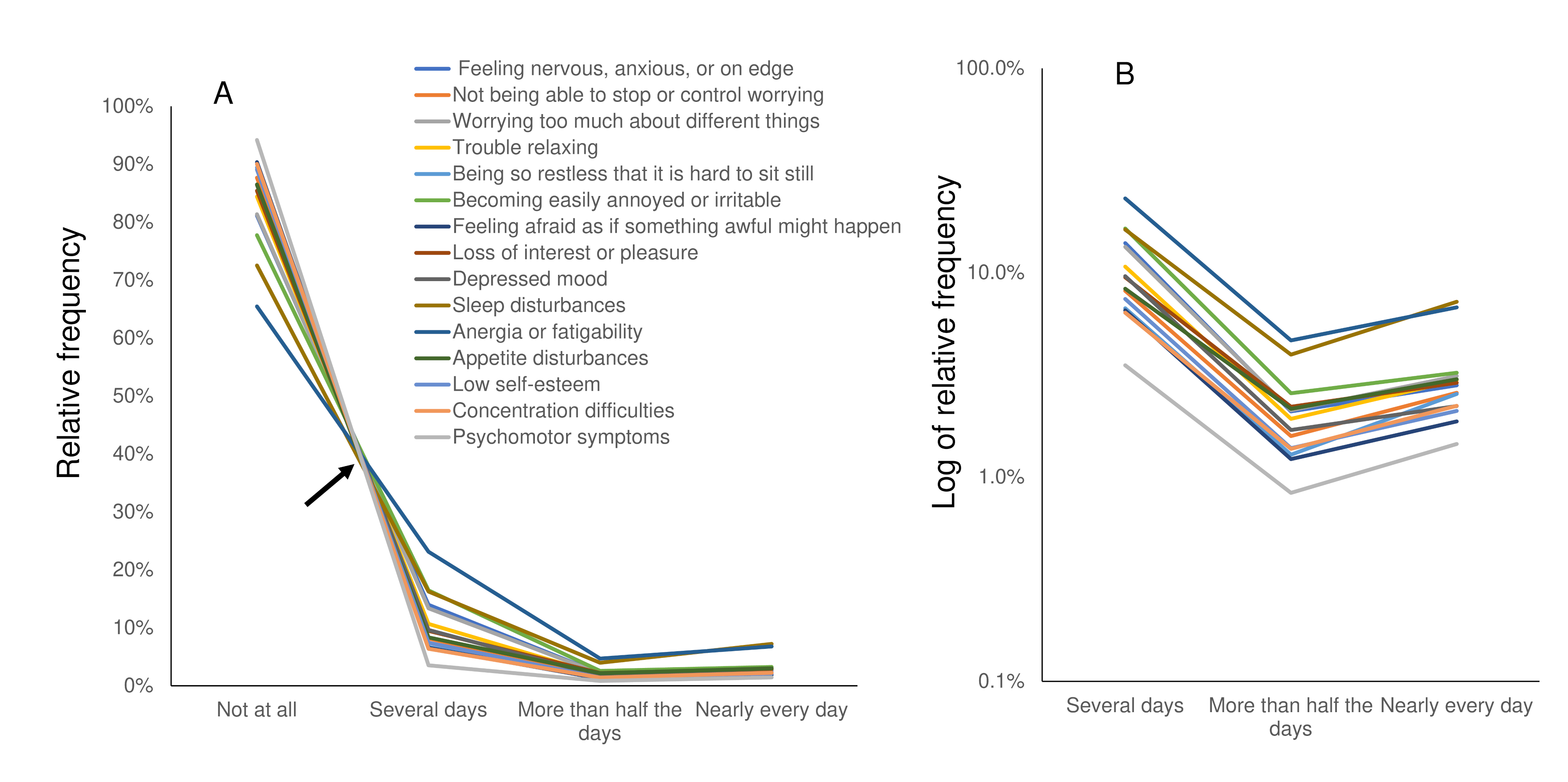

Supplement: Supplementary file 2 — Additional file 2: Fig. S1. Responses to the 15 items of the Patient Health Questionnaire-8 and the Generalized Anxiety Disorder-7 in males. Responses by males to the 15 items are presented using a normal scale (A) and a logarithmic scale (B). A As indicated by the arrow, the line graphs of the 15 items appear to cross at a single point between “not at all” and “several days.” The lines decrease in synchrony from “several days” to “more than half the days,” before increasing in synchrony from “more than half the days” to “nearly every day.” [file 40359_2021_657_MOESM2_ESM.tif]

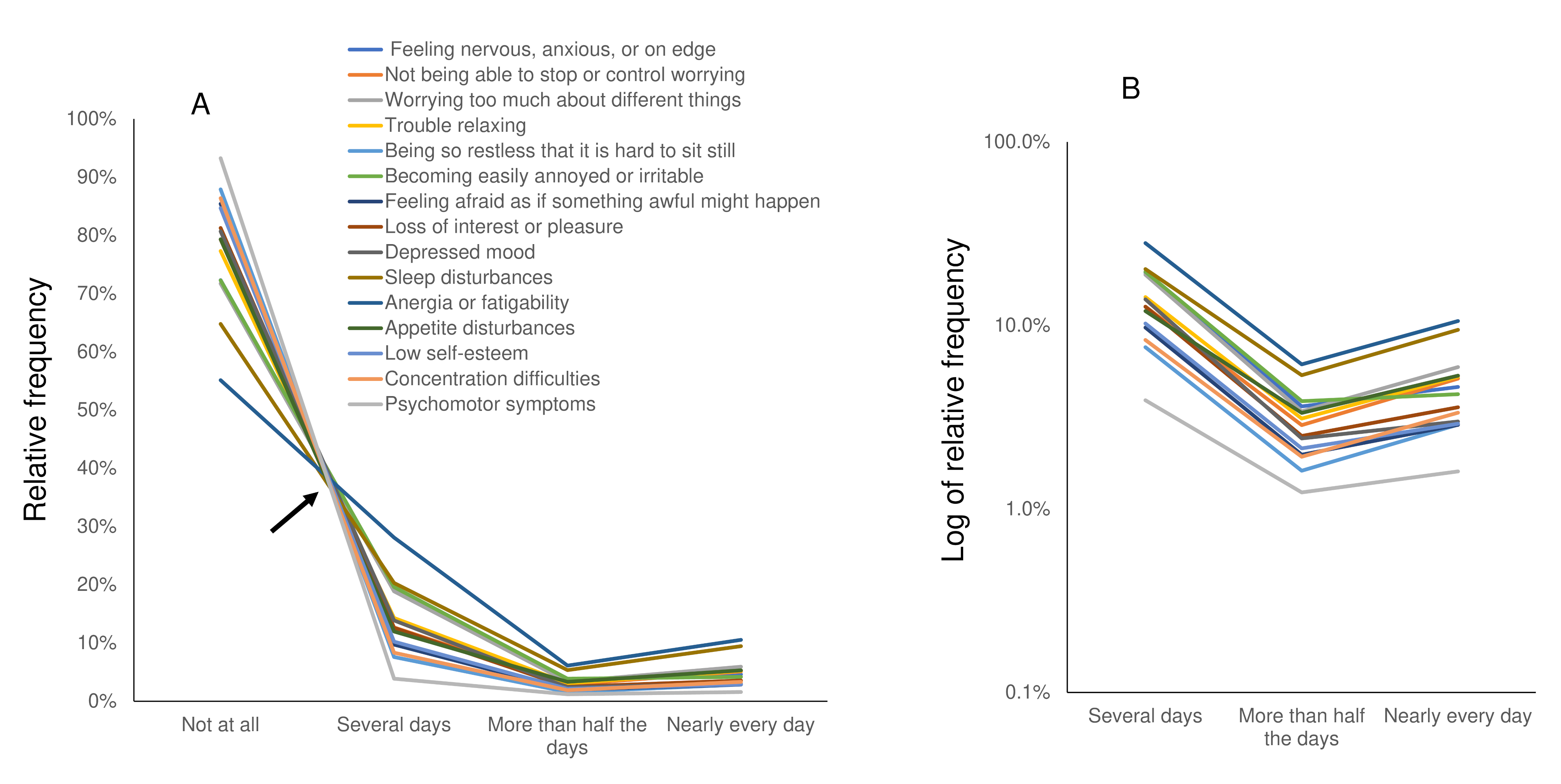

Supplement: Supplementary file 3 — Additional file 3: Fig. S2. Responses to the 15 items of the Patient Health Questionnaire-8 and the Generalized Anxiety Disorder-7 in females. Responses by females to the 15 items are presented using a normal scale (A) and a logarithmic scale (B). A As indicated by the arrow, the line graphs of the 15 items appear to cross at a single point between “not at all” and “several days.” The lines decrease in synchrony from “several days” to “more than half the days,” before increasing in synchrony from “more than half the days” to “nearly every day.” [file 40359_2021_657_MOESM3_ESM.tif]
